# Baby Y anastomosis, the journey towards total arterial complete myocardial revascularization

**DOI:** 10.1016/j.xjtc.2023.03.014

**Published:** 2023-04-01

**Authors:** Vignesh Ratnaraj, Amit K. Tripathy, Asen Ivanov, Sarah Scheuer, Shivanad Gangahanumaiah, John Goldblatt, Robin Brown

**Affiliations:** Department of Cardiothoracic Surgery, The Royal Melbourne Hospital, Parkville, Victoria, Australia

**Keywords:** total arterial grafting, Y-grafting, coronary bypass surgery

**Figure:**
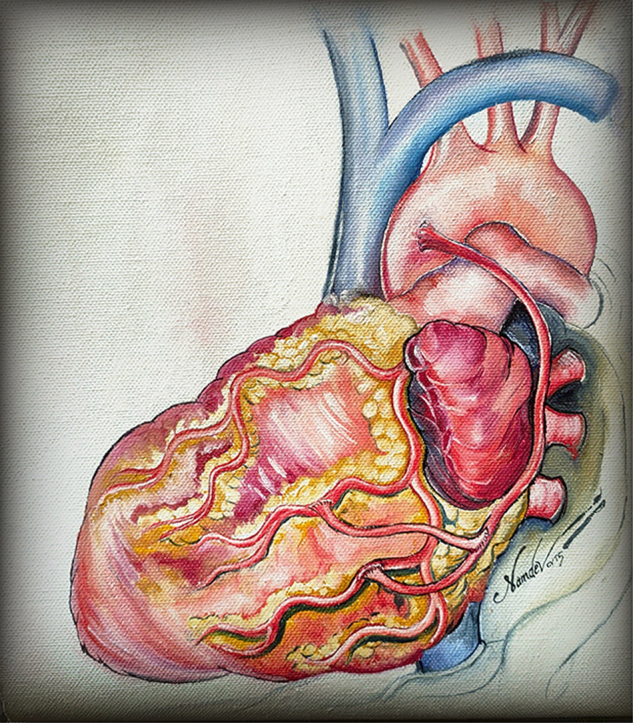
Baby Y anastomosis of the marginal arteries to achieve total arterial revascularization.

**Central Message**

Baby, or mini, Y anastomosis helps achieve total arterial complete myocardial revascularization by utilizing redundant segments of the arterial conduits.

Understanding coronary artery disease and its optimal management has been the subject of continued research and debate during the past 6 decades. Coronary artery bypass grafting (CABG) is the most frequently performed cardiac operation globally^1^ and is a class I indication in triple-vessel coronary artery disease and diabetes.^2^ Surgical revascularization strategies primarily involve the use of conduits such as internal thoracic arteries (ITAs), radial arteries (RAs), gastroepiploic arteries, and saphenous veins. These are constructed in various configurations. In situ grafting for ITAs and gastroepiploic arteries, aortocoronary grafts, and lastly Y or T anastomoses in a side-to-side manner, are in vogue. Due to lack of consensus surrounding methodology of CABG, the decision ultimately rests on the operating surgeon.

At The Royal Melbourne Hospital in Australia, we have been using a strategy of total arterial complete myocardial revascularization since the 1990s with left ITA (LITA) and RAs being the predominant conduits.^3^ Since 2010, we have evolved a technique of using redundant segments of the left RA (LRA), right RA (RRA), and/or LITA to perform baby Y grafts. Baby Y is defined as the redundant segment of a parent conduit used for coronary revascularization that is ≤5 cm. This segment subsequently serves as an additional conduit and is utilized for revascularizing a target coronary artery as a Y graft. The baby Y segments have their proximal ends coming off an aortacoronary graft (parent limb) in a side-to side manner and distal end anastomosed to a target coronary artery in an end-to-side fashion.^4^^,^^5^

The primary advantages of this method include constructing 4 to 6 distal anastomoses using 2 to 3 arterial conduits (LITA, LRA, and RRA) and avoiding the need for a sequential graft.^6^^,^^7^ This benefits the surgeon by providing extra conduits to revascularize the additional target vessels while utilizing the redundant segment of the arterial conduits that would otherwise be discarded or would require harvesting of an additional arterial or venous conduits. It also reduces the need to construct any additional anastomoses on the LITA, which is only grafted to a significantly stenosed left anterior descending artery (LAD) and is considered sacrosanct in CABG. This additionally avoids extra manipulation of the ascending aorta. This helps avoid an extra conduit (usually a vein) and an additional wound.^8^ The possible disadvantages of this technique may be an increase in crossclamp time by 5 to 10 minutes per baby Y graft and an initial learning curve. In our observation, this can be buried against the greater goal of achieving complete revascularization. In this article, we aim to illustrate this technique in various operative scenarios and provide technical, pictographic, and angiographic references for the same.

## Surgical Technique

**Figure 1:**
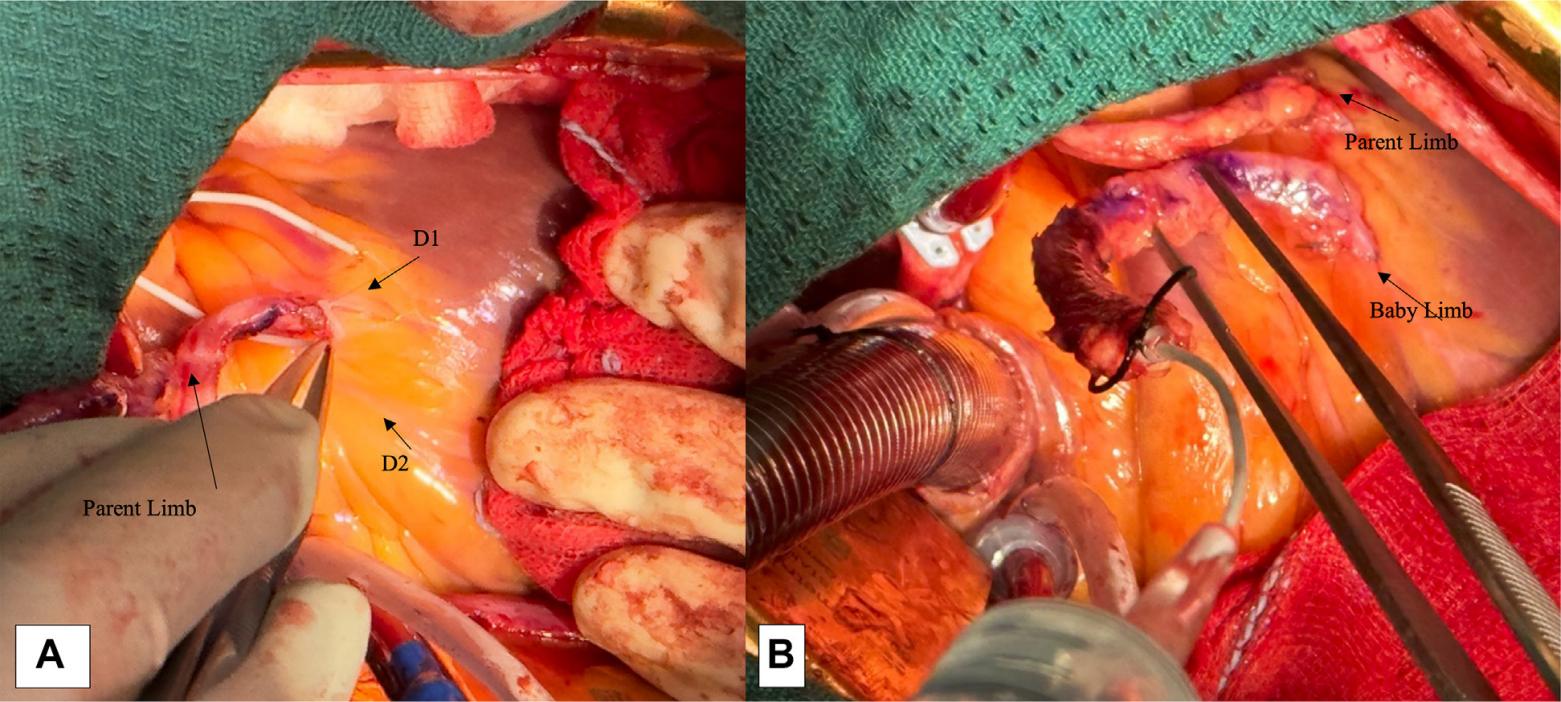
Preparing the baby Y limb. A, Distal anastomosis with the parent conduit to the target coronary artery (D1 in this case). B, Redundant length (future baby Y limb) obtained after measuring the parent limb up to the aorta.

While utilizing 1 arterial conduit each for LAD (in situ), left circumflex (aortocoronary), and dominant right coronary targets (aortocoronary), the baby Y anastomoses are primarily constructed with redundant segments of the radial or thoracic conduits. They branch off proximally from the corresponding aortocoronary segment of the RAs and can be used to revascularize the target coronary arteries on the way. In this manner, grafting the acute marginal and posterior left ventricular branch on the right side and bypassing obstructed obtuse marginal branch, ramus, and diagonal arteries on the left side can be achieved with maximum of 3 arterial conduits. The length of the baby Y can vary from 3 to 5 cm and we have many times used the redundant segments to construct 2 to 3 additional anastomoses in a baby Y fashion (Figure 1).

### Preoperative Angiographic Assessment and Choice of Conduits

Most of the operation is already planned before putting skin to knife in terms of targets and conduits. A good assessment of preoperative coronary angiograms helps in planning the surgical strategy. We usually utilize the LITA for the LAD as a solo graft. A single radial artery, more often than not, is enough to revascularize the rest of the targets on the left side using just 1 parent and multiple baby Y anastomoses, using the redundant segments of the LITA and the RA.^9^^,^^10^ These redundant segments are thoroughly inspected to ensure that they are of good quality—not injured or spasmed—before committing to use them as conduits. Similarly, when the right coronary artery system needs to be bypassed, another RA is harvested. We have never needed more than 3 conduits to perform coronary artery surgery using this technique even when performing 8 to 10 anastomoses.

We continue to use instrumented RRAs as our third conduit believing they would have similar or better long-term patency in comparison to a vein graft. All instrumentation during the past 10 years has been ultrasound-guided and occur quite distally on the RRA. We have a protocol of using preoperative ultrasound to assess quality of the RRA before using it to assess luminal patency, size, dissection, and collateral circulation.^11^

### Construction of a Baby Y Anastomosis

We strongly emphasize that the farthest target in an area must get an arterial conduit constructed as the parent aortocoronary bypass. The baby Y grafts are created out of this parent trunk to the target coronary arteries that are to be bypassed and that lie along the way, such as diagonals, ramus, and obtuse marginal branch on the left, and acute marginal posterior left ventricular branch on the right side.

#### Measurements and proximal end of aortocoronary graft

The top end of the aortocoronary conduit, which serves as a parent limb for the baby Y grafts, are usually performed after completion of all the planned baby Y anastomoses on the target coronary arteries, including completion of the proximal and distal end of the Y limbs.

After the distal anastomosis of the parent graft is complete, the parent conduit is brought up to the ascending aorta and measured for future proximal aortocoronary anastomosis. The redundant segment of this is excised and preserved and is what will serve as a future baby Y limb. For the left system, the parent conduit is brought up along the pericardium, posterior to the left atrial appendage, and measured to the aorta. For the right-sided grafts, it is conventionally anastomosed to the posterior descending artery and brought up along the curve of the right atrium to the ascending aorta.

Once the parent conduit is measured, the extra segment is trimmed and fashioned. The parent conduits are placed in their final position. The baby Y segments are then placed on the parent conduit with the heart gently retracted and the future site of proximal anastomosis marked with a medium clip. At the same time, a decision is made on the length of the Y limb based on the proximity of the target artery to the parent limb (Figure 1). In case of a plan to construct more than 1 baby Y anastomosis from the same parent limb (and shortage of conduits), only a small segment of the redundant segment can be used, whereas the rest serves as conduit for the next anastomosis. This essentially means the length of the baby Y limb is based on the placement and availability of conduits for completion of all the planned anastomoses. Hence, the proximal anastomosis of the Y limb on the parent conduit can be adjusted accordingly.

#### Distal

**Figure 2:**
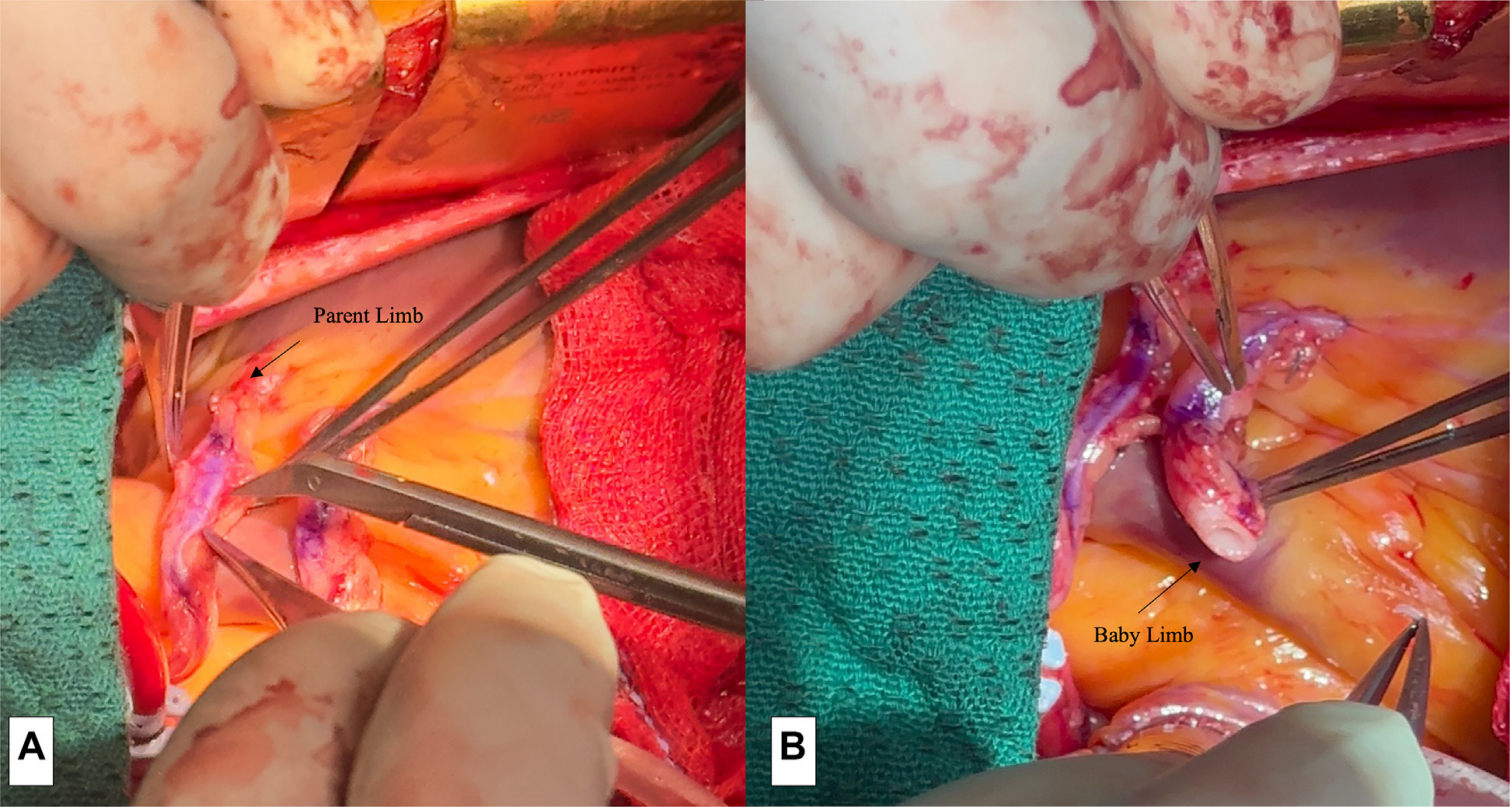
Constructing the proximal end of the Y limb. A, Showing the arteriotomy made on the side of the parent limb to construct the Y anastomosis. B, Proximal end of the baby Y limb fashioned on its side to form a side to side anastomosis.

The distal anastomosis is constructed in a usual manner using 7–0 polypropylene sutures in an end-to-side fashion (Figure 2).

#### Proximal

**Figure 3:**
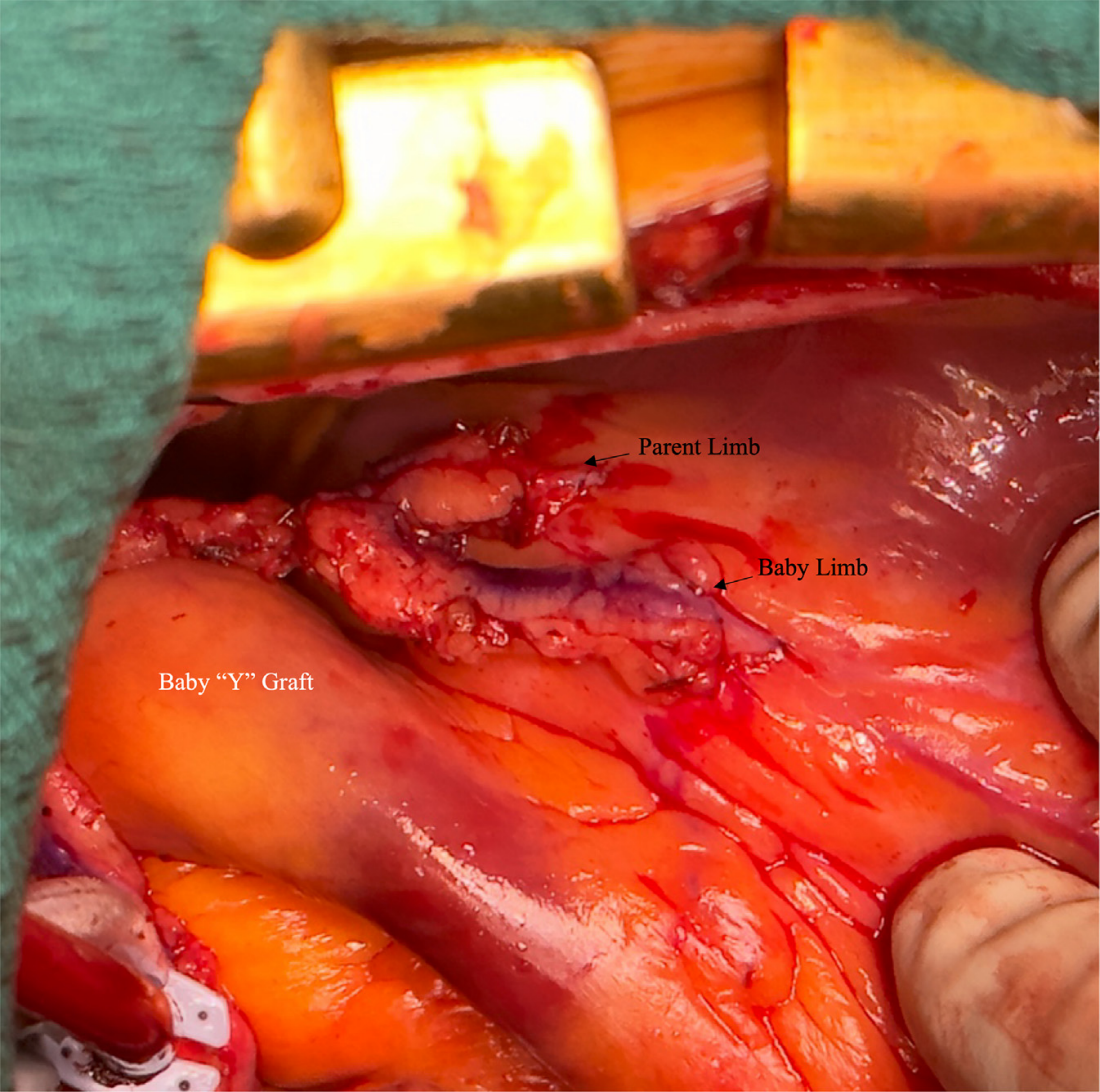
Showing completed Baby Y graft flowing to the first and second diagonal branches of the left anterior descending artery.

Because the proximal end of the parent limb has not been constructed, the heart can be appropriately positioned using packs and the parent limb pulled up anteriorly to perform the proximal end of the baby Y limb. In keeping with fluid dynamics, we perform an arteriotomy on the parent limb on the side rather than on the top, and similarly fashion the proximal end of the baby Y limb for an appropriate anastomosis (Figure 3).

### Intraoperative Details

It takes 15 to 20 minutes of crossclamp time to complete 1 baby Y anastomosis for the senior authors of this article. This may be 5 minutes more than performing an aortocoronary graft (proximal and distal). With appropriate myocardial protection this does not appear to be concerning when considering the greater picture to achieve complete arterial revascularization.

### Postoperative Angiograms

**Figure 4:**
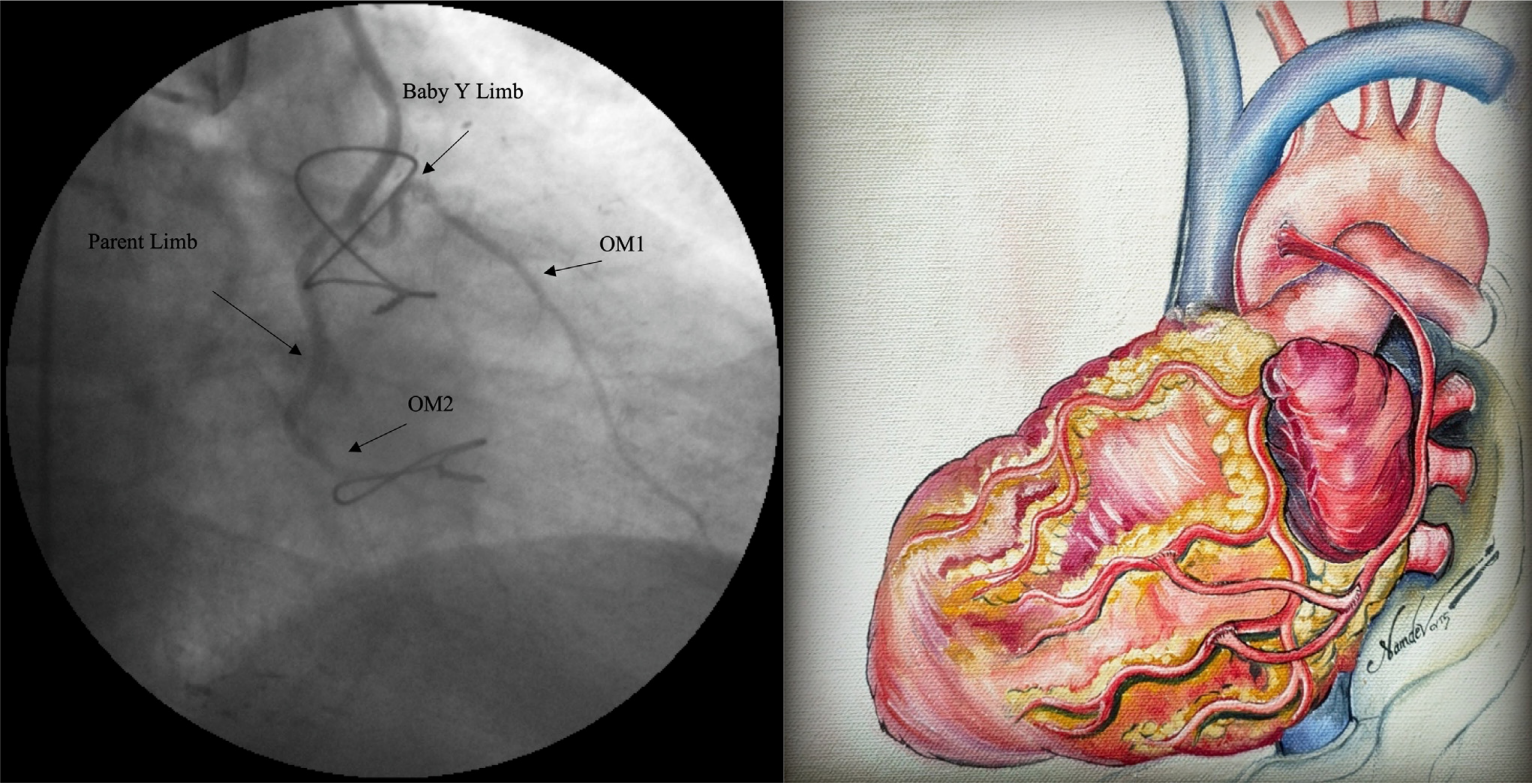
Angiogram showing a patent baby Y graft to the first and second obtuse marginal (OM) arteries 10 years after the index surgery (*left*). Artist impression of the same baby Y graft to OM1 and OM2 (*right*).

As a part of a larger study, some of those who underwent baby Y grafts underwent postoperative coronary angiograms. The images in Figure 4 reinforce our belief in this technique that simulates physiology or natural arterial blood supply.

## Discussion

Although CABG is the most frequently performed cardiac surgical operation globally, there is complete lack of consensus about number of anastomoses; choice of conduits; technique of CABG (off vs on pump); graft configurations (in situ, sequential, or Y grafts); and, in recent days, thoracotomy versus sternotomy to obtain similar results.^1^^,^^12^ Challenges also include availability of conduits because a majority of patients have 1 or more of the following conditions: old age, diabetes mellitus, obesity, peripheral arterial disease, varicose veins, chronic kidney disease, or sternal injury from cardiopulmonary resuscitation. The combination of CABG with additional procedures such as a redo setting, valve replacements, and myectomy also dictate grafting strategies; so does the grade of left ventricular dysfunction, degree of target artery stenosis, and recency of surgery in relation to acute coronary syndrome.^13^^,^^14^

It is always in the best interest of the patient to have total arterial complete myocardial revascularization. This strategy necessitates bypassing every obstructed major or branch coronary artery (with ≥70% obstruction) that is ≥1.25 mm.^15^^,^^16^ The size of the coronary artery catheter (5 or 6 Fr) usually guides preoperative decision making in judging the size of the target coronary artery and determining ability to bypass it, as does the distribution of the coronary artery. It is frequently seen that a major branch coronary artery has independent, significant stenosis in its anterior and posterior branches that need separate bypasses.

The patency results of LITA-to-LAD grafts have been demonstrated time and again.^17^^,^^18^ This serves as the basis for contemporary coronary artery surgery. Constructing an additional anastomosis on the LITA in addition to the LAD may not be necessary and does not serve the best interest of the patient. The choice of the second and third conduits vary. There is enough evidence to suggest that the arterial conduits outperform the venous ones in the longer run.^3^^,^^4^ The benefits of Y grafting are that it completely avoids recurrent aortic manipulation and can be configured in numerous ways to reach target vessels regardless of how the native vessels lie.^6^^,^^19^ Angiographic data also support the concern that obstruction or compromise of graft flow to the distal segment of a sequential graft, can not only affect flow to the second anastomosis, but also compromise that of the primary anastomosis when compared with Y grafting.^7^

The idea of a baby Y graft is that it replicates both the physiology and physics of a coronary artery. Major coronary artery branches into major limbs supply blood to the heart; similarly, baby Y grafts are small branches coming off a parent limb that is anastomosed proximally to the aorta and distally to a major branch coronary artery. The baby Y limbs are obtained from redundant parent limbs that otherwise are discarded while another conduit is harvested to bypass the same vessel—or a sequential anastomosis performed. The list of advantages is long and disadvantages few.

Our technique is a simple, safe, and reproducible approach in sync with the notion of total arterial complete myocardial revascularization that utilizes minimal conduits to revascularize obstructed coronary arteries with the aid of baby Y arterial segments. This not only replicates the physiology and flow dynamics of native coronary branching, but also avoids using a vein graft, which often is the case due to lack of conduits. A larger angiographic patency study will ultimately be needed to validate the potential benefits of this method.

### Limitations

This technique has evolved in our institution over the past 10 to 12 years. We are yet to ascertain the angiographic 20-year results of these baby Y anastomoses. Having said that, it is an extrapolation of the T or Y grafts that have been performed routinely over the past 3 decades. Secondly, we primarily utilize cardiopulmonary bypass to perform CABG. This technique utilizes a lot of fine measurements to result in a perfect anastomosis in terms of lie and length of the baby Y limbs. Using this technique for off-pump CABG—a routine procedure in many centers across the world—should be dealt with using caution.

### Conflict of Interest Statement

The authors reported no conflicts of interest.

The *Journal* policy requires editors and reviewers to disclose conflicts of interest and to decline handling or reviewing manuscripts for which they have a conflict of interest. The editors and reviewers of this article have no conflicts of interest.
